# PREVENTION OF SYMPTOMATIC NEUROMA BY USING SYNTHETIC CONDUITS IN FINGER AMPUTATION STUMPS

**DOI:** 10.1590/1413-785220243201e283207

**Published:** 2025-04-07

**Authors:** ERICK YOSHIO WATAYA, DEBORAH BERNARDO LOPES, DIOGO KENZO TAKAZONO, MARIANA MIRANDA NICOLOSI PESSA, RENATO POLESE RUSIG, LUIZ SORRENTI, LUCIANO RUIZ TORRES, TENG HSIANG WEI, MARCELO ROSA DE REZENDE, RAMES MATTAR

**Affiliations:** 1 Universidade de Sao Paulo, Faculdade de Medicina, Hospital das Clinicas HC-FMUSP, Departmento de Ortopedia e Traumatologia DOT, Sao Paulo, SP, Brazil.

**Keywords:** Amputation, Traumatic, Fingers, Neuroma, Nerve Conduit, Sensory Recovery, Amputação traumática, Dedos, Neuroma, Conduite de Nervo, Recuperação Sensorial

## Abstract

**Objective:**

Compare the formation of symptomatic neuromas in patients submitted to digital amputations, with and without nerve conduits (Neurolac^®^ ), and sensitivity return.

**Methods:**

Prospective, case-control study, including 14 patients with digital amputations (total of 17 fingers) whose conduits were used on the ulnar or radial side, while the contralateral side was used in the same patients as control. The Tinel test, Semmes-Weinstein monofilament, and two-point discrimination tests were evaluated at one week, two weeks, one month, three months, and six months postoperatively.

**Results:**

Using nerve conduits (Neurolac^®^) *in* digital nerve amputation stumps had statistical significance (p = 0.04) in preventing pain due to symptomatic neuroma at the end of six months after digital regularization.

**Conclusion:**

There is a favorable trend towards using conduits as prophylaxis of symptomatic neuroma formation since the nerves in which they were used showed fewer clinical signs of neuroma formation six months after surgery. ***Level of evidence II, Prospective comparative study.***

## RESUMO

**Objetivo:**

Comparar a formação de neuroma sintomático em pacientes submetidos a amputações digitais com e sem uso de neurotubo (Neurolac^®^) e avaliar retorno de sensibilidade.

**Métodos:**

Estudo prospectivo, caso-controle. Amostra de 14 pacientes com amputações digitais (total de 17 dedos) onde foram utilizados conduítes no lado ulnar ou radial, enquanto o contralateral no mesmo paciente foi usado como controle. Avaliados quanto ao teste de Tinel, monofilamenteo de Semmes-Weinstein e discriminação de 2 pontos com 1 semana, 2 semanas, 1 mês, 3 meses e 6 meses de pós-operatório.

**Resultados:**

o uso de conduíte neural (Neurolac^®^) em cotos de amputação de nervo digital apresentou significância estatística (p = 0,04) na prevenção de dor decorrente de neuroma sintomático ao final de 6 meses da regularização digital.

**Conclusão:**

Há tendência favorável ao uso de conduíte como profilaxia à formação de neuroma sintomático, já que, os nervos em que foram utilizados, apresentaram menos sinais clínicos de formação de neuroma após 6 meses da cirurgia. **Nível de evidência II, Estudo prospectivo comparativo.**

## INTRODUCTION

Traumatic digital amputations are frequent injuries, especially in manual workers, due to the use of circular saws, and can cause a high rate of functional disabilities*.* ^1^

In the event of impossibility or failure of replantation, the approach to traumatic amputation of fingers is to regularize the stump to a lower bone level that allows primary closure, or the use of flaps that allow better coverage of the injury, without the need for shortening.

The most common procedure regarding radial and ulnar digital nerve stumps is neurectomy of each nerve under traction, made in a single section, and with a cold blade. This would prevent the formation of neuromas near the surgical scar, which can generate neuropathic pain and discomfort for manual functions*.* ^(^ ^2^ ^)-(^ ^4^

In some cases, even after an appropriate traction neurectomy symptomatic neuromas appear, which cause functional impairment to the patient.

New techniques for the reconstruction of a complex neurological injury, in which there is segmental loss, or distance between the nerve stumps, enable the use of nerve conduits as an alternative to autografts*.* ^(^ ^5^

Nerve conduits, or nerve tubes, are tubes usually made of synthetic silicone or polysaccharides, absorbable or not, which can serve as a guide for nerve regeneration in cases of injury with a distance between the stumps*.* ^(^ ^6^ They create an appropriate microenvironment for axonal growth, which can generate good functional results^7^ ^)-(^ ^8^ *,*and do not bring morbidity to the donor area, such as autogenous grafts*.* ^(^ ^9^ ^)-(^ ^10^ However, its use is restricted to cases in which there is a small distance between the nerve stumps.^*(*^ ^11^ ^)-(^ ^12^

They can also be used in the treatment of post-traumatic neuroma as a support to contain the neuroma inside a cul-de-sac, which reduces neuropathy pain due to friction with the scar, with satisfactory results*.* ^(^ ^13^ ^)-(^ ^15^ Koch (2011) had satisfactory results in the prevention of post-traumatic neuroma by transposing the nerve stump into a vein segment.^*(*^ ^16^

However, in cases of digital amputations, injuries with great potential to generate neuroma, it is still uncertain whether the technique of restraining the nerve within the synthetic nerve conduit would better prevent symptomatic neuroma formation, compared to traction neurectomy alone.

Self-absorbing synthetic caprolactone conduit (Neurolac^®^) is an option for injury reconstruction with spacing between stumps, with good results and without risk of immunogenic reaction*.* ^(^ ^17^ ^)-(^ ^20^

The study aims to compare the formation of symptomatic neuroma in patients undergoing digital amputations with and without the use of neurotube (Neurolac®️) and to evaluate return sensitivity using the Semmes-Weinstein monofilament test and the two-point discrimination test in nerves with and without conduit.

## METHODS

This study was conducted at the IOT - HC - FMUSP and the sample consisted of patients coming from the emergency room or hospitalized. The study was approved by the Research Ethics Committee in Human Beings (IOT-HC-FMUSP) under Protocol No. 4,707,488.

This was a prospective, randomized, double-blind, case-control study.

### Inclusion criteria

- Patients with injuries in Verdan Flexor Zones one or two (distal to pulley zone A1).
- Patients with single or multi acute digital amputations with no indication for reimplantation.
- Patients who evolved with loss of reimplantation of one or more digits and regularization need.
- Application of a free informed consent form.

### Exclusion criteria

- Loss of patient follow-up.
- Digital nerves not found in the stump.
- Complex, segmental, multi-level injuries.
- Patients with inclusion criteria for the study were randomized, by an observer out of the study, to apply the conduit to the radial or the ulnar digital nerve. Informed consent form was applied for the surgical procedure.

Epidemiological, injury descriptive, and trauma mechanism were collected from the patient data and placed in an Excel table.

- Patient data: Name, age, RGHC, comorbidities, occupation, dominant hand.
- Injury data: Trauma mechanism, injured digital, regularization level, injury zone (one or two Verdan), conduit use (in radial or ulnar digital nerve).

The outcome analyzed was formation or absence of symptomatic neuroma in digital amputation stumps after placement or not of nerve conduit.

### Surgical technique

**Figure 1:**
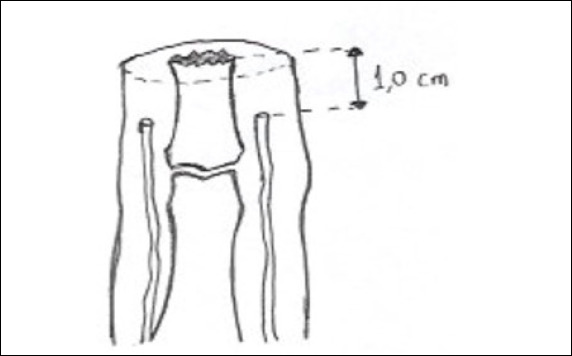
Amputation stump after bone regularization and cold neurectomy

After bone regularization and before primary or via locoregional flaps closure, the ulnar and radial digital nerves will be dissected proximately to the ideal point for neurectomy, approximately 1.0 centimeters below the bone level (Figure 1).

The neurectomy will be done with a 15-scalpel blade, through a single cut.

**Figure 2:**
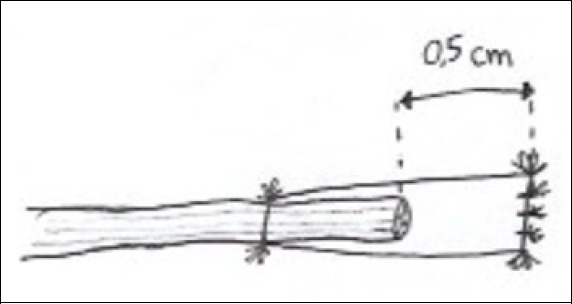
Nerve ending sutured with conduit in cul-de-sac.

Then, one of the radial or ulnar digital nerves will be capped with the conduit, with about 0.5 centimeters of distance between the nerve stump ending and the conduit ending (Figure 2).

**Figure 3:**
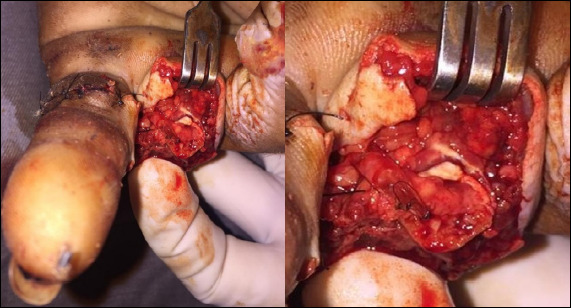
Clinical image demonstrating neurotube in the digital nerve.

The conduit will be sutured to the nerve epineurium with two stitches at 180 degrees, with 8.0 Nylon suture thread (Figure 3). The conduit ending will also be sutured with 8.0 Nylon thread to restrain the nerve ending in cul-de-sac.

Then, primary or via locoregional flaps closure will be performed, as indicated in each case.

### Postoperative

The patients were discharged in the first postoperative period, with simple dressings, without the need for immobilization.

They used prophylactic antibiotics for at least one week.

The first return visit occurred after one week for surgical wound evaluation, in which there was already a referral to occupational therapy for evaluations.

Return in the second week for a new evaluation and stitches removal.

From then on, clinical evaluations were performed at one, three, and six months postoperative and involved:

- Search of symptomatic neuroma via pain presence in shock by the Tinel test, both to ulnar and radial sides of the amputation stump.
- Search for sensitivity feedback via Semmes-Weinstein monofilament test, and two-point discrimination, also for the radial and ulnar sides of the stump.

### Statistical analysis

Descriptive statistics were used to describe the sample. The normality of the numerical variables (age, number of errors in the two-point discrimination test, and thickness of the Semmes-Weinstein monofilaments) was tested using the Shapiro-Wilk test, which did not indicate normality for all the variables analyzed. Therefore, the median, minimum (min) and maximum (max) values were used to describe the numerical variables. 

Absolute (n) and relative (%) frequencies were used to describe categorical variables (Verdan’s zone, surgical procedures, injury mechanisms, Tinel sign). 

The differences in the Tinel sign occurrence (Yes vs. No) were tested for each follow-up period and for the last valid data of each patient via the McNemar test. The positive Tinel occurrence was considered as a positive outcome in cases in which Tinel sign was present equal to or greater than the contralateral nerve sign (Table 1).

**Table 1:** Evaluation of Tinel sign occurrence.

Outcome	Description	Example
Negative	Tinel sign lower than in contralateral nerve	Tinel sign of the case group lower than the control group
Positive	Tinel sign equal to or greater than in contralateral nerve	Equal positive Tinel sign between case and control

The Wilcoxon test was used to compare the thickness of the Semmes-Weinstein monofilament and the number of errors in the two-point discrimination test in each follow-up period and for the last valid data of each patient. 

The p < 0.05 value was used as statistical significance for all comparisons. All analyses were performed using the statistical software Stata 14 (StataCorp, College Station, TX, EUA). 

## RESULTS

The study had an initial sample of 14 patients with a median age of 33.0 years (minimum eight and maximum 67 years). All patients had the right hand as dominant and three of them injured more than one finger, totaling 17 observations at the baseline of the study. Most injuries occurred in Verdan zone two (82.3%). At the end of follow-up, only four patients presented valid data, totaling a follow-up loss of 76.5%. 

Table 2 shows the surgical procedures performed on the patients. Regularization of the second finger in FP was the most prevalent procedure in the sample studied (23.3%), followed by thumb regularization in IF (11.8%), third finger regularization in FM (11.8%), and fourth finger regularization in FM (11.8%).

**Table 2:** Types of surgical procedures performed (n = 17).

Procedure	Occurrence	%
Thumb regularization in IF	2	11.8%
Thumb regularization in FP	1	5.9%
Thumb regularization in MCF	1	5.9%
Regularization of the second finger in FD	1	5.9%
Regularization of the second finger in FP	4	23.3%
Regularization of the second finger in IFP	1	5.9%
Regularization of the second finger in IFP	2	11.8%
Regularization of the second finger in FP	1	5.9%
Regularization of the fourth finger in FM	2	11.8%
Regularization of the fourth finger in IFP	1	5.9%
Regularization of the fifth finger in IFP	1	5.9%

FP: proximal phalanx; FM: middle phalanx; FD: distal phalanx; MCF: metacarpal phalangeal joint; IFP: proximal interphalangeal joint; IFD: distal interphalangeal joint; IF: interphalangeal joint of the thumb.

**Figure 4:**
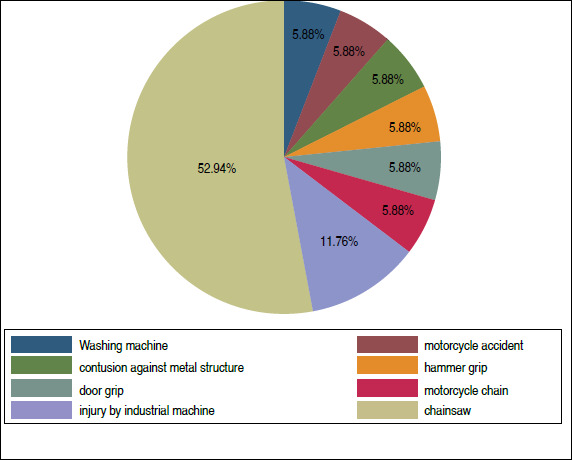
Injury mechanism

Figure 4 shows the injury mechanisms reported by the patients. Most injuries (52.9%) were caused by circular saw handling, followed by injuries by industrial machinery (11.8%).

Of the 17 observations, nine had the use of conduit on the ulnar digital nerve and eight on the radial digital nerve. Follow-up of the patients occurred after one and two weeks and one, three and six months after the surgical procedure. Table 3 shows the occurrence of Tinel for each conduit using group over the follow-up period and using the last valid data from each patient.

**Table 3:** Tinel occurrence for each conduit using group during the six months of follow-up.

	Follow-up period										Last valid data (n = 34)	
	1 week (n = 28)		2 weeks (n = 24)		1 month (n = 18)		3 months (n = 16)		6 months (n = 8)			
Conduit use	n (%)	p*	n (%)	p*	n (%)	p*	n (%)	p*	n (%)	p*	n (%)	p*
Yes	14 (100%)	0.31	10 (83.3%)	0.15	5 (55.5%)	0.18	6 (75.0%)	1.00	**0 (0%)**	**0.04**	12 (70.6%)	0.25
No	13 (92.9%)		12 (100%)		8 (88.9%)		6 (75.0%)		**4 (100%)**		15 (88.2%)	

* = p value for McNemar’s accurate test; n = number of observations mixing the Case and Control groups.

Meaningful differences were observed only in the six-month follow-up period, with Tinel positive occurring only on the side without conduit use (p = 0.04). Table 4 shows the descriptive data and the comparison of the use of Semmes-Weinstein monofilaments between the groups in each of the follow-up periods and for the last valid data of each patient. The comparisons identified no important difference between the conduit using groups.

**Table 4:** Thickness of the Semmes-Weinstein monofilaments used in each conduit using group in the follow-up periods and for the last valid data of each patient.

	Follow-up period						Last valid data	
	1 month		3 months		6 months			
Conduit use	Median	p*	Median	p*	Median	p*	Median	p*
	(min - max)		(min - max)		(min - max)		(min - max)	
Yes	0.20	0.63	0.20	0.54	0.20	0.31	0.05	0.19
	(0.05 - 0.20)		(0.05 - 0.20)		(0.05 - 0.20)		(0.05 - 0.2)	
No	0.20		0.05		0.05		0.05	
	(0.05 - 4.0)		0.05		(0.05 - 0.05)		(0.05 - 4.0)	

* = p value for the Wilcoxon test for differences between the conduit using groups.

Table 5 shows the median, minimum, and maximum values of errors obtained in the two-point discrimination test for each conduit using group in the one, three, and six-month follow-up periods and for the last valid data of each patient. No difference was seen between the conduit using groups.

**Table 5:** Number of errors obtained in the two-point discrimination test for each conduit using group in the follow-up periods and for the last valid data of each patient.

	Follow-up period						Last valid data	
	1 month (n = 14)		3 months (n = 7)		6 months (n = 4)			
Conduit use	Median	p*	Median	p*	Median	p*	Median	p*
	(min-max)		(min-max)		(min-max)		(min-max)	
Yes	4.5	0.94	5.0	0.93	3.5	0.84	4.0	0.93
	(0 - 9.0)		(1.0 - 9.0)		(1.0 - 8.0)		(0 - 10)	
No	6.0		3.0		2.5		3.0	
	(0 - 10.0)		(0 - 9.0)		2.0		(0 - 9.0)	

* = p value for the Wilcoxon test for differences between the conduit using groups.

## DISCUSSION

The formation of symptomatic digital neuroma is frequent and can cause limitations. Van der Avoort et al. (2013), in a retrospective cohort study, concluded the incidence of people with symptomatic neuroma after traumatic digital amputation is higher than people undergoing primary neurorrhaphy.^*(*^ ^21^ Also, patients with symptomatic neuroma have higher rates of reoperation than those with asymptomatic neuroma. 

Vlot et al. (2018), also in a retrospective study, analyzed 1083 patients who underwent review of digital amputation stumps aiming to evaluate painful neuroma.^*(*^ ^22^ He concluded that about one in 15 patients will develop symptomatic neuroma after amputation and approximately 50% will require surgical review. Because of that, the development of methods that prevent the formation of neuroma that generates pain, or limitation of function after digital amputations, would reduce the risk of reoperations and functional impairment to the patient.

In the face of a traumatic digital amputation, some techniques are described in nerve stump management and in the prevention of symptomatic neuroma appearance. Swanson et al. (1977), Thomsen et al. (2010), and Gould et al. (2013) described good results in the prevention of symptomatic neuroma with nerve restriction within neurotubes.^*(*^ ^13^ ^)-(^ ^15^

Thomsen et al. (2010) retrospectively evaluated the formation of symptomatic neuroma in digital nerves and common digital nerves after digital amputations in 10 patients, using collagen conduit.^*(*^ ^14^ After an average period of 18 months, it showed good results in 50% of cases in relation to the two-point discrimination. There was a decrease in sensitivity to light touch and protective sensitivity in about 80% of the cases and no cases of pain recurrence.

Gould et al. (2013) also evaluated the use of synthetic conduits in nerve stumps at the ankle and foot level.^*(*^ ^15^ In a retrospective study, out of a total of 69 nerves evaluated, 43% showed total improvement in pain after the use of the neurotube.

Lans et al. (2020), in a retrospective study, evaluated 33 symptomatic digital neuromas surgically treated through repair (39%), and simple excision with (30%) or without (30%) implantation of the stumps.^*(*^ ^23^ Cases in which repair or reconstruction was performed had better results in terms of pain improvement.

All these studies had good results, favorable to the use of some type of conduit that would prevent the onset of neuroma, but they were retrospective studies.

The use of synthetic nerve conduits, in addition to the good results in reestablishing neural connection and restraining the neuroma, thus improving symptoms, also has a low immunogenic response*.* ^(^ ^17^ ^)-(^ ^20^

Shin et al. (2009) functionally and histologically compared the use of autograft with several other types of nerve conduits in rat nerve gaps.^*(*^ ^18^ The neurotube consisting of caprolactone was like the autograft in the functional return in 10mm segmental defects of the sciatic nerve. Luis et al. (2007) was also favorable to caprolactone as a component of conduits regarding functional return responses and axonal regeneration rate.^*(*^ ^24^

Bertleff et al. (2005) used the caprolactone substitute as a guide for nerve regeneration in segmental injuries compared to other neural reconstruction techniques, also presenting favorable results for the use of conduit in transects of nerves in the hand.^*(*^ ^20^

In a series of 12 cases, Costa Serrão de Araújo et al. (2017) demonstrated that caprolactone conduits are also favorable in reestablishing sensory response in segmental nerve injuries of up to 25mm.^*(*^ ^17^

This study aimed to prospectively analyze whether the use of caprolactone conduit (Neurolac^®^) can alleviate or prevent the formation of symptomatic neuroma in nerve stumps of digital amputations.

The sample of this study consisted of young adults, with a median age of 33 years, and the right hand affected. The amputations were mainly caused by a circular saw (52.9%), mainly affecting Verdan zone 2 (82.3%) of the index finger (23.3%). These data confirm the age group most likely to suffer severe trauma or amputations in the upper limb caused by circular saws in the national context and reinforce the need for correct and safe use during manual work. 

Facing a single digital amputation in Verdan zone two, the success of reimplantation is questionable, due to the high chance of generating stiffness of the finger to be reimplanted, due to the high number of structures to be rebuilt in this region. This may be one of the reasons why the number of regularizations at this level was higher.

The presence of Tinel sign, which causes excruciating pain to nerve percussion, indicating the presence of neuroma, was positive in most observations of the first week after surgery, regardless of the group. Only one patient had a larger Tinel sign on the conduit side compared to the control; however no statistical difference was seen (p = 0.31).

In the second week and first month, the positive Tinel sign was more prevalent on the control side, and in the third month, the occurrence of Tinel was similar between both groups. Despite this higher prevalence of Tinel sign in the control group in the second week and first month, no statistical difference was seen (p > 0.05). 

Only in the sixth postoperative month this comparison was statistically important, and in this period, in all cases in which conduits were not used, the Tinel sign was present.

The last valid data per patient in relation to Tinel sign showed that it was positive in 88.2% of the nerves without the conduit; versus 70.6% of nerves with conduit.

Regarding the sensitivity evaluation by the Semmes-Weinstein monofilament test, there was a favorable trend for nerves without conduit at three and six months. However, there was no statistical importance in any period. And, in the last valid data per patient, the nerves with and without conduit presented equal medians.

In the two-point discrimination test, patients had a lower rate of errors on the conduit side only in the first month. At three and six months after surgery, the median number of errors favored the side without the conduit. On the other hand, there was a higher rate of correct answers over time, which indicates an improvement in the return of sensitivity on both sides. No statistical importance was observed.

This study presented some limitations. The low number of patients and the small number of fingers evaluated negatively favored the analysis, interfering with the results.

Loss to patient’s follow-up was also a limiting factor that interfered with data analysis. Some determining factors were, because these are injuring whose treatment is definitive and that presents rapid improvement, providing an early return to routine activities, the patients often missed the return for evaluations. The global COVID-19 pandemic may also be a negative factor that has contributed to the loss to several patients’ follow-up.

Based on epidemiological data of the study, it is possible to ratify the importance of proper handling of circular saws and occupational safety measures for manual workers to avoid injuries, often mutilating.

An important difference with p = 0.04, favorable to the use of conduit, occurred at the end of the six-month follow-up in the Tinel test evaluation. However, the number of patients who maintained follow-up during this period was low.

The group analysis of the last valid data per patient favored the conduit use in the Tinel test; similar on the sides with and without conduit in the monofilament test; and favored the non-conduit side in the two-point discrimination test.

Even in the absence of statistical importance in most analyses, this study points to a favorable trend towards the use of conduit as a prophylaxis for the formation of symptomatic neuroma, since the nerves in which they were used showed fewer clinical signs of neuroma formation. A study with greater inclusion of patients and a lower rate of follow-up loss may elucidate the issue in favor or not of the use of conduit for nerve stumps after digital amputation.

## CONCLUSION

The use of nerve conduit (Neurolac^®^) in digital nerve amputation stumps showed statistical importance in the prevention of pain resulting from symptomatic neuroma at the end of six months of digital regularization. However, there was no statistical difference in the sensitivity analysis at the end of the period.
